# On the impact of improved dose calculation accuracy in clinical treatment planning for superficial high-dose-rate brachytherapy of extensive scalp lesions

**DOI:** 10.1016/j.phro.2024.100673

**Published:** 2024-11-16

**Authors:** Giulio Rossi, Vasiliki Peppa, Mark Gainey, Michael Kollefrath, Tanja Sprave, Panagiotis Papagiannis, Dimos Baltas

**Affiliations:** a Division of Medical Physics, Department of Radiation Oncology, Medical Center – University of Freiburg, Faculty of Medicine, University of Freiburg, German Cancer Consortium (DKTK), Partner Site DKTK, Freiburg, Germany; b Medical Physics Laboratory, Medical School, National and Kapodistrian University of Athens, Greece; c Department of Radiation Oncology, Medical Center – University of Freiburg, Faculty of Medicine, University of Freiburg, German Cancer Consortium (DKTK), Partner Site DKTK, Freiburg, Germany

**Keywords:** HDR ^192^Ir mold brachytherapy, TG-43, MC simulation, MBDCA

## Abstract

TG-43-based dose calculations disregard tissue heterogeneities and finite scatter conditions, prompting the introduction of model-based dose calculation algorithms (MBDCAs) to improve accuracy in high-dose-rate (HDR) brachytherapy. This study evaluated the effectiveness of MBDCAs over TG-43 in HDR ^192^Ir brachytherapy of extended scalp lesions. Treatment planning dose calculations were compared with Monte Carlo (MC) data. TG-43 exhibited a dose overestimation ranging from 10% to 23% as the distance from the implant increased, while a better agreement from 2% to 6% was observed between the MBDCA and MC, supporting the adoption of MBDCAs for dose calculations in broad scalp lesions.

## 1 Introduction

Despite the literature supporting the implementation of model-based dose calculation algorithms (MBDCAs) in brachytherapy treatment planning [1], the TG-43 formalism [2] remains the preferred approach due to its simplicity and practicality. TG-43 relies on the superposition of single-source dose distributions in an unbounded water geometry, yet factors like tissue heterogeneities, finite patient dimensions, and the presence of needles/applicators are disregarded. Although site-specific studies have not shown significant clinical effects on average [3], [4], TG-43 may be less accurate in certain applications, such as High-Dose-Rate (HDR) intraoperative and surface brachytherapy, where source dwell positions lie near the edge of a bounded heterogeneous geometry surrounded by air, limiting full scatter conditions.

Raina *et al.* [5] reported a TG-43 dose overestimation in intraoperative HDR ^192^Ir treatments due to the lack of full scatter conditions, which increased with prescription distance (up to 13 % of the prescription dose for 1.5 cm), leading to the suggestion of bolus use or dose prescription amendment. Boman *et al.* [6] compared the TG-43 and MBDCA options of a commercial treatment planning system (TPS) in HDR ^192^Ir brachytherapy superficial treatments, indicating a significant TG-43 dose overestimation when full scatter conditions do not apply, with deviations up to 15 % for larger molds. TG-43 dose overestimations were also evident in the comparison between the TG-43 and MBDCA options of a commercial TPS by Scherf *et al.* [7] and Placidi *et al.* [8] for HDR ^192^Ir treatments of perinasal skin tumor and eyelid cancer, respectively. To avoid underdosage, Placidi *et al.* [8] suggested the use of bolus when a MBDCA is not available.

The Monte Carlo (MC) studies by Vjiande *et al.* [9] and Granero *et al.* [10] showed differences less than 5 % between TG-43 and MC in geometries simulating HDR ^192^Ir skin treatments. Based on these works, the American Brachytherapy Society (ABS) recommends that a bolus is not needed for HDR skin brachytherapy [11]. Since rather small applicators were simulated in these studies however, and in light of the aforementioned experimental and clinical works showing clinically significant TG-43 inaccuracies, this recommendation may be too general, and further investigation on the impact of improved dose calculation accuracy for cases with larger applicators would be beneficial.

Such investigation was the purpose of the present work, comparing TG-43 to a collapsed cone superposition MBDCA and MC simulation for a HDR ^192^Ir mold brachytherapy treatment of scalp lymphoma. These cases are interesting due to the large extent of the lesions requiring custom molds with pronounced curvature and a large number of dwell positions.

## 2 Materials and methods

### 2.1 Treatment planning

This study involved a HDR surface brachytherapy application for the scalp performed at the Radiotherapy Clinic of the University Medical Center Freiburg (Germany), using the ^192^Ir mHDR-v2 source model in a microSelectron HDR v3 afterloader (Elekta AB, Sweden). The prescribed dose was 36 Gy administered in 18 fractions. The clinical target volume (CTV) covered almost the entire scalp (Fig. S1a in Supplementary Material). An individualized mold (Fig. S1b, c in Supplementary Material) was shaped to station the source in 840 dwell positions using 26 6F plastic needles (Elekta AB). The mold was made from overlapping layers of a thermoplastic material (Efficast 2.4 mm maxi, Orfit Industries, Belgium, thickness: 2 mm, mass density: 1.13 g/cm^3^). Radiolucent buttons (Elekta AB), also made of thermoplastic material (acrylonitrile butadiene styrene, mass density 1.03 – 1.07 g/cm^3^) were used to fix the needles within the mold.

The source dwell positions were activated based on Al-markers. Prescription depth (3–5 mm tissue depth) was defined using dose points generated 10 mm from the activated source dwell positions. TG-43-based dose planning was performed using the OncentraBrachy V4.3.0.410 (Elekta AB) TPS.

### 2.2 Retrospective dose calculations

The treatment planning data was exported in DICOM RT format and imported into OncentraBrachy V4.6.0.026 TPS (Elekta AB). Dose re-calculations were performed using both the TG-43 and the Advanced Collapsed cone Engine (ACE) [12] dose calculation algorithms of the TPS. ACE calculations were performed in both high-HA and standard-SA accuracy level [12]. Dose was calculated as dose to water in water *D_w,w_* with full scatter by TG-43, and as dose to medium in medium *D_m,m_* in the ACE calculations, using a 1x1x1 mm^3^ grid. For the ACE calculations, the density assignment was HU-based, while the material composition was assigned based on the TG-186 report [1]. In short, CTV and normal skin were defined as mean skin, bones as cortical bone, brain, eyes, and optic nerves as mean male soft tissue, lenses as eye lens, and the mold as water, since Scherf *et al.* [7] showed negligible variation of the dose parameters related to the planning target volume (PTV) for different typical mold materials with respect to water. The calculation time was approximately 10 min for TG-43, and approximately 2–3 days and 3 h for HA and SA ACE, respectively.

### 2.3 MC dose calculations

Simulations were performed using MCNP v6.1 [13] with input files prepared using BrachyGuide [14] to parse the information from the treatment plan in DICOM RT format. A summary of methods used in the simulation [15] is in Table S1 in Supplementary Material. Differences between ACE and MC results due to the different material composition assignment schemes (*e.g.* the mold was assigned as soft tissue in MC) are negligible for the ^192^Ir energies when the effect of density is ruled out [16], [17].

### 2.4 Dose calculation comparison

TG-43 and ACE were compared with MCNP in terms of local dose differences on a voxel-by-voxel basis, and dose-volume histogram (DVH) indices for the CTV and the OARs (Table S2 in Supplementary Material). Since, generally, the accepted degree of accuracy for clinical dose calculation in HDR brachytherapy is within 5 % [1], [9], [18], differences above this threshold were deemed as clinically significant.

### 2.5 Ethics

Informed consent for publication was obtained in written form from a 64 years old male with a primary cutaneous germinal center lymphoma (follicular B-cell lymphoma) of the scalp.

## 3 Results

**Fig. 1:**
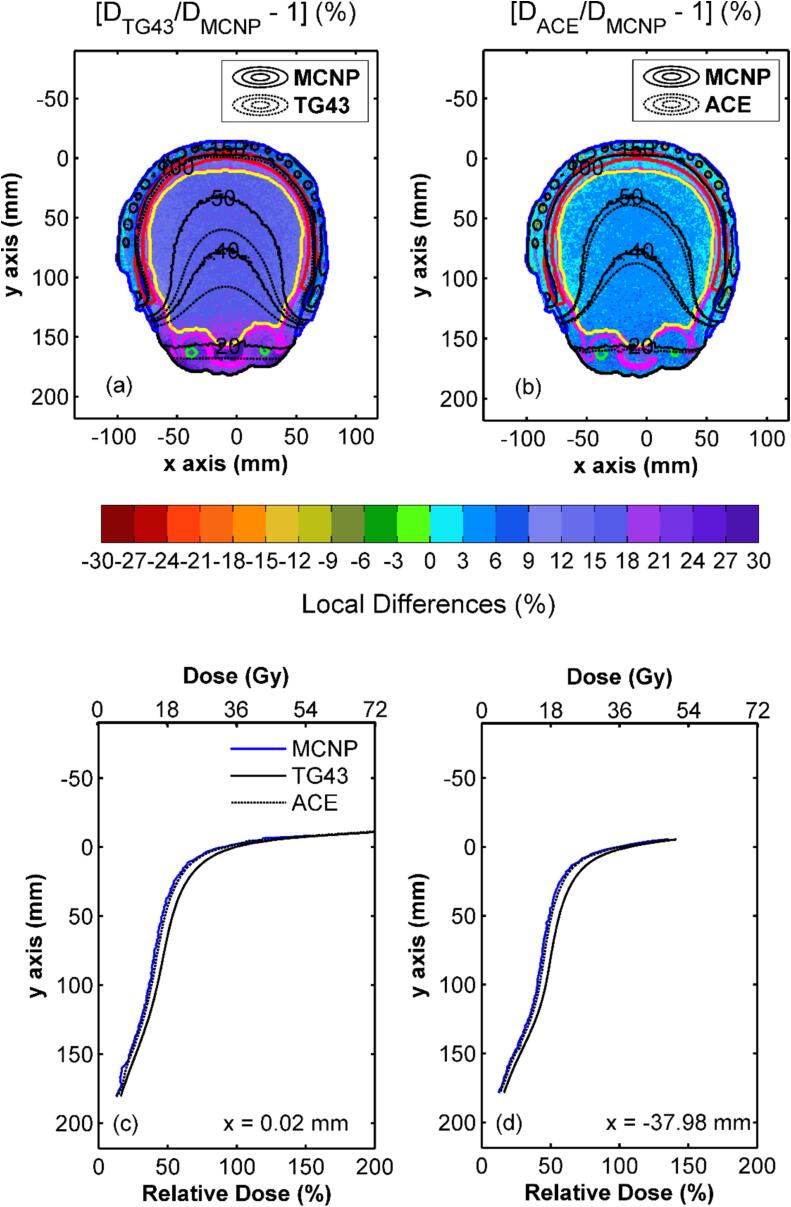
Colormap representations of the local dose differences between (a) TG-43 and (b) HA ACE with MCNP results on an axial slice, with selected percentage isodose lines (20 %, 40 %, 50 %, 100 %, 150 %) superimposed (red contour: CTV, black contour: external, blue contour: mold, magenta contour: bones, yellow contour: brain, green contour: eyes). Corresponding results are shown on a sagittal plane in Fig. S2 of Supplementary Material. Dose profiles along the y-axis calculated on the same axial slice at (c) x  = 0.02 mm and (d) x  = -37.98 mm using MCNP, HA ACE and TG-43 are also presented. (For interpretation of the references to colour in this figure legend, the reader is referred to the web version of this article.)

The voxel-based comparison in Fig. 1 shows a considerable TG-43 dose overestimation relative to MCNP data, that exceeded type A MC uncertainty (max. 2 % *k* = 1, see Table S1 in Supplementary Material) for the vast majority of voxels, and increased with distance from the implant from approximately 4 % (within the mold) up to a maximum of 23 % within the lenses. HA ACE and MCNP were found in excellent agreement within the CTV and bones, with differences of approximately 2 %. This agreement deteriorated slightly with increasing distance from the implant, but remained within 5 % within the brain and the lenses. Slightly higher differences, up to 6 %, were only observed in low dose regions such as the eyes and the optic nerves.

The comparisons in Fig. S3 in Supplementary Material further highlight the systematic TG-43 overestimations. Median local dose differences of TG-43 relative to MCNP varied from +9.6 % for the CTV to +22.4 % for the right lens. Normal skin was the only structure presenting considerable negative differences near the patient chin and throat. The median local dose differences between HA ACE and MCNP were more evenly distributed, ranging from +2.2 % for the CTV and the bones, to +6.1 % for the right optic nerve. Although HA ACE also exhibited a slight tendency to overestimate the dose with respect to MCNP, the observed differences within the CTV and the most proximal OARs to the implant were mostly comparable with type A MC uncertainty.

Table 1 summarizes DVH indices calculated for the CTV and the OARs using TG-43, SA and HA ACE and MCNP, and corresponding percentage differences using the latter as reference. Within the CTV, both TG-43 and ACE overestimated the minimum dose delivered at given percentages of the CTV. This overestimation was however up to 7 % for TG-43, 1.7 % for HA ACE and 1.8 % for SA ACE. Similarly, the minimum dose delivered at different volumes of the normal skin exhibited differences greater than 5 % between TG-43 and MCNP and up to 1.1 % between ACE and MCNP. Differences of DVH indices between TG-43 and MCNP were greater for the remaining OARs, exceeding 20 % for OARs at relatively increased distance from the implant such as the eyes and eye lenses. Corresponding differences between ACE and MCNP remained under 6 %. These findings agree with the DVH comparison in Figs. S4 and S5 of Supplementary Material. Data in Table 1 also suggest a close agreement between HA and SA ACE results (see the corresponding voxel-based comparison in Fig. S6 in Supplementary Material).

**Table 1:** TG-43, HA and SA ACE and MCNP-based DVH indices for the CTV and critical structures.

Structure	DVH indices	(a) MCNP	(b) TG43	(c) ACE (HA)	(d) ACE (SA)	% Differences^1^		
						(b)-(a)	(c)-(a)	(d)-(a)
CTV	D_90_ (%)	60.8	67.9	62.5	62.6	7.1	1.7	1.8
	D_98_ (%)	30.6	37.1	32.0	32.1	6.5	1.5	1.5
	D_2_ (%)	113.3	120.4	114.1	114.3	7.1	0.9	1.0
Normal Skin	D_0.1 cm_^3^ (Gy)	33.9	35.7	33.9	34.0	5.4	0.0	0.2
	D_1 cm_^3^ (Gy)	29.6	31.4	29.8	29.8	5.9	0.5	0.6
	D_10 cm_^3^ (Gy)	25.4	27.4	25.7	25.7	7.9	0.9	1.1
	D_max_ (Gy)	49.7	51.6	47.2	47.1	4.0	−5.0	−5.1
Bones	D_0.1 cm_^3^ (Gy)	33.7	36.5	33.7	33.7	8.5	0.1	0.2
	D_1 cm_^3^ (Gy)	31.7	34.7	31.9	31.9	9.6	0.7	0.8
	D_50_ (Gy)	9.3	10.5	9.5	9.5	12.2	1.9	2.0
	D_mean_ (Gy)	21.6	24.3	22.0	22.0	12.6	2.1	2.2
	D_max_ (Gy)	36.6	38.9	36.1	36.4	6.3	−1.3	−0.6
Brain	V_50_ (%)	21.3	24.4	22.0	22.1	14.7	3.3	3.6
	D_mean_ (Gy)	15.6	18.0	16.2	16.2	15.5	3.9	4.2
Right Eye	D_2 cm_^3^ (Gy)	6.3	7.6	6.6	6.6	21.0	5.4	5.3
	D_mean_ (Gy)	5.7	7.0	6.1	6.1	21.7	5.7	5.5
	D_max_ (Gy)	7.6	9.1	7.9	7.9	19.2	4.0	4.0
Left Eye	D_2 cm_^3^ (Gy)	5.8	7.1	6.1	6.1	22.1	5.9	5.8
	D_mean_ (Gy)	5.4	6.6	5.7	5.7	22.5	6.1	6.0
	D_max_ (Gy)	7.1	8.4	7.4	7.3	19.4	4.3	3.2
Right Lens	D_0.01 cm_^3^ (Gy)	5.3	6.5	5.6	5.5	22.6	5.1	4.6
	D_mean_ (Gy)	5.0	6.1	5.2	5.2	22.6	4.9	4.7
	D_max_ (Gy)	5.5	6.6	5.7	5.7	19.9	3.2	2.8
Left Lens	D_0.01 cm_^3^ (Gy)	5.1	6.3	5.3	5.3	23.7	4.6	4.9
	D_mean_ (Gy)	4.7	5.8	5.0	4.9	23.4	5.1	4.7
	D_max_ (Gy)	5.2	6.4	5.4	5.4	21.9	3.9	3.9
Right Optic Nerve	D_0.01 cm_^3^ (Gy)	8.7	10.1	9.1	9.1	16.4	4.3	4.9
	D_mean_ (Gy)	7.6	9.0	8.1	8.1	18.3	6.0	6.5
	D_max_ (Gy)	8.9	10.3	9.2	9.3	15.1	3.1	3.8
Left Optic Nerve	D_0.01 cm_^3^ (Gy)	9.1	10.5	9.6	9.6	15.1	5.3	4.9
	D_mean_ (Gy)	8.0	9.4	8.5	8.5	17.4	5.6	5.7
	D_max_ (Gy)	9.5	10.7	10.0	9.7	12.3	4.9	1.9

1 For the *D_x_* (%) and *V_x_* (%): % Dose Differences = (b) – (a) or (c) – (a) or (d) – (a) For the *D_x_* (Gy): % Dose Differences = 100×{(b)/(a) – 1} or 100×{(c)/(a) – 1} or 100×{(d)/(a) – 1}.

## 4 Discussion

A TG-43 dose overestimation was observed in a HDR ^192^Ir skin brachytherapy application characterized by a large lesion with pronounced curvature, treated with a large number of catheters/source dwell positions. The potentially significant overestimation for the CTV (about 10 % or the equivalent of two fractions) is attributed to the TG-43 overestimation of scatter dose on account of its full scatter geometry assumption [19], [20]. This effect even counterbalanced the increased mass energy absorption of bone over water [21], [3], and led to a TG-43 dose overestimation in bone. TG-43 dose overestimation increased with distance from the implant due to the increase of relative importance of scatter over primary dose combined with the disregard of increased attenuation in bone. It should be mentioned however that, apart from the lenses, where the maximum dose values were close to the dose tolerance limit [22], [23], the significant TG-43 dose overestimation observed within the eyes and optic nerves was not clinically relevant, as toxicity in these OARs is anticipated at substantially higher dose levels [22], [23].

ACE was found in close agreement with MCNP, especially within the CTV, the normal skin and the bones (within 2 %). This agreement deteriorated slightly at larger distances from the implant, with ACE also tending to slightly overestimate dose. This is probably due to the dimensions of the phantom used for the calculation of the dose deposition kernel for multiple scatter being greater than those in the treatment geometry [24] combined with ray artifacts evident in ACE calculations at larger distances from the source [25], [26], [27].

The latter are artifacts due to the angular discretization employed by both currently available MBDCAs to enhance calculation speed, in the form of dose overshoot along the finite number of directions used. They are more pronounced for small numbers of dwell positions at points where scatter dose gradient is high and primary dose is small. They can be mitigated with the increase of directions and voxel size at the expense of computational time and potential volume averaging effects, respectively. Given the increased number of dwell positions considered in this work, SA and HA ACE calculations achieved comparable accuracy with the former taking considerably less time.

Scherf *et al.* [7] and Placidi *et al.* [8] reported smaller differences between TG-43 and ACE within the target, while Vijande *et al.* [9] and Granero *et al.* [10] reported smaller differences between TG-43 and MC simulation compared to our findings, yet significantly smaller molds were used in these studies. Differences observed between TG-43 and MCNP within the CTV in this work are in accordance with those observed by Raina *et al.* [5] at a 5 mm prescription distance. Boman *et al*. [6] showed a TG-43 dose overestimation within the CTV by approximately 16 % compared to Acuros, which was relatively larger than the 10 % overestimation observed between the TG-43 and MCNP results of this work, probably due to the different loading patterns in the two studies.

The above findings suggest that recommendations against using bolus in HDR skin brachytherapy may be too general [11], and there have been studies suggesting specific bolus thickness [8] albeit for specific applications of relatively reduced lesion size. For increased treated lesion sizes, such as in this work, the bolus required to achieve acceptable dose calculation accuracy using TG-43 might be patient-specific and/or impractical.

According to our results, an alternative approach is the use of a MBDCA for individualized, patient-specific dose planning, provided that the MBDCA is carefully commissioned [28] and first used in parallel to TG-43 to ascertain the validity of prescription for typical cases.

## CRediT authorship contribution statement

**Giulio Rossi:** Conceptualization, Methodology, Validation, Formal analysis, Investigation, Resources, Data curation, Writing – original draft, Visualization. **Vasiliki Peppa:** Methodology, Software, Validation, Formal analysis, Investigation, Resources, Data curation, Writing – review & editing, Visualization. **Mark Gainey:** Resources, Writing – review & editing. **Michael Kollefrath:** Resources. **Tanja Sprave:** Supervision, Project administration. **Panagiotis Papagiannis:** Supervision, Writing – review & editing. **Dimos Baltas:** Conceptualization, Investigation, Resources, Supervision, Writing – review & editing.

## Declaration of competing interest

The authors declare that they have no known competing financial interests or personal relationships that could have appeared to influence the work reported in this paper.
