# Supplementary material for: On the impact of improved dose calculation accuracy in clinical treatment planning for superficial high-dose-rate brachytherapy of extensive scalp lesions
Source: Phys Imaging Radiat Oncol. 2024 Nov 16;32:100673. doi: 10.1016/j.phro.2024.100673 (PMC11616074; doi:10.1016/j.phro.2024.100673)
Supplement: Supplementary Data 1 [file mmc1.pdf]

On the impact of improved dose calculation accuracy in clinical treatment planning for superficial high-dose-rate brachytherapy of extensive scalp lesions

Supplementary Material

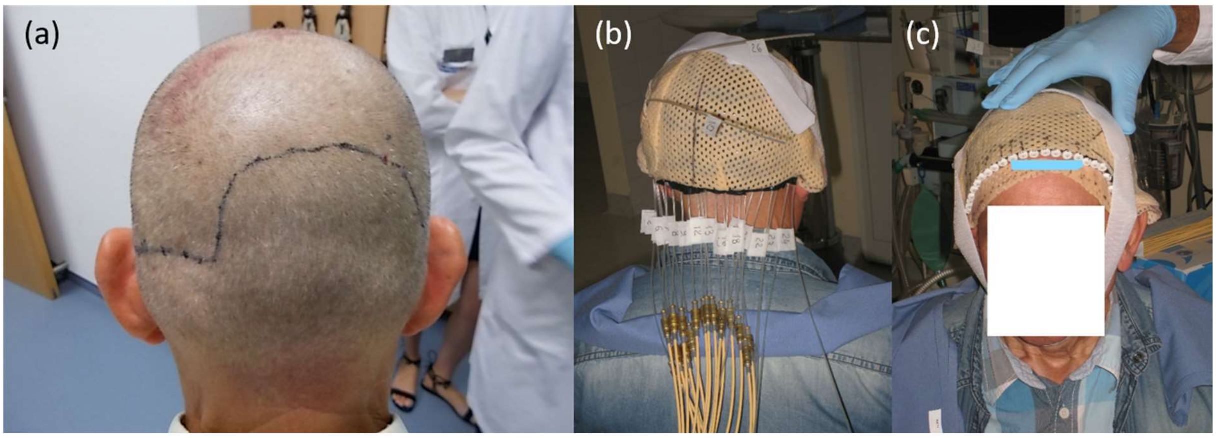

Fig. S1. Photographs depicting CTV delineation (a) and the applied mold (b, c) used in this work.

Table S1
Main features of the MC simulations.

Item	Description	References
MC code	MCNP v6.1. Input file generated with BrachyGuide v1.0	[1], [2]
Hardware	PC model Dell Precision Rack 7910; CPU: 2x Intel® Xeon® CPU E5-2670 v3 @ 2.30 GHz (24 cores); RAM: 128 GB DDR4.	
Source description	Phase-space file of the ^{192}Ir source mHDR-v2 with energy, position, and direction of photons emitted from the source. A transformation was applied to each photon position and direction from the phase-space file to match with the source dwell position and the orientation within the needle. Electron emissions were neglected.	[2], [3]
Patient geometry	Rectangular lattice. The size of the lattice element was equal to the voxel size of the imported CT images. Dimensions of the geometry equal to the field-of-view.	[2], [3]
Density assignment	Voxel-by-voxel using a default CT calibration of HU vs density.	[2], [3]
Material composition assignment	Using the obtained mass density, the elemental composition of each voxel was determined based on a look-up table of 23 human tissue composition bins.	[4]
Cross sections	EPDL97 photon cross sections.	[5]
Scored quantities	Absorbed dose to medium in medium $D_{m,m}$ approximated with kerma to medium in medium $K_{m,m}$ (Gy) in each voxel of the geometry using the MCNP F6 scoring tally.	[2], [3], [6]
Dose grid	0.55x0.55x1 mm^3	
CPU time (range)	~ 20 days.	
Type A uncertainty	About 1 % for voxels less than 5 cm away from the implant, increasing to 2 % at the boundaries of the geometry.	
Type B uncertainty	≤ 0.1%	[3]

Table S2

Description of the DVH indices used for the CTV and the OARs.

Notation	Description
D_x (%)	The minimum percentage of the planning aim dose delivered at given percentages of a structure
$D_{x\,\mathrm{cm}^3}$ (Gy)	The minimum dose delivered at given volumes of a structure
D_x (Gy)	The minimum dose delivered at given percentages of a structure
V_x (%)	The percentage of the structure receiving dose greater than given percentages of the planning aim dose

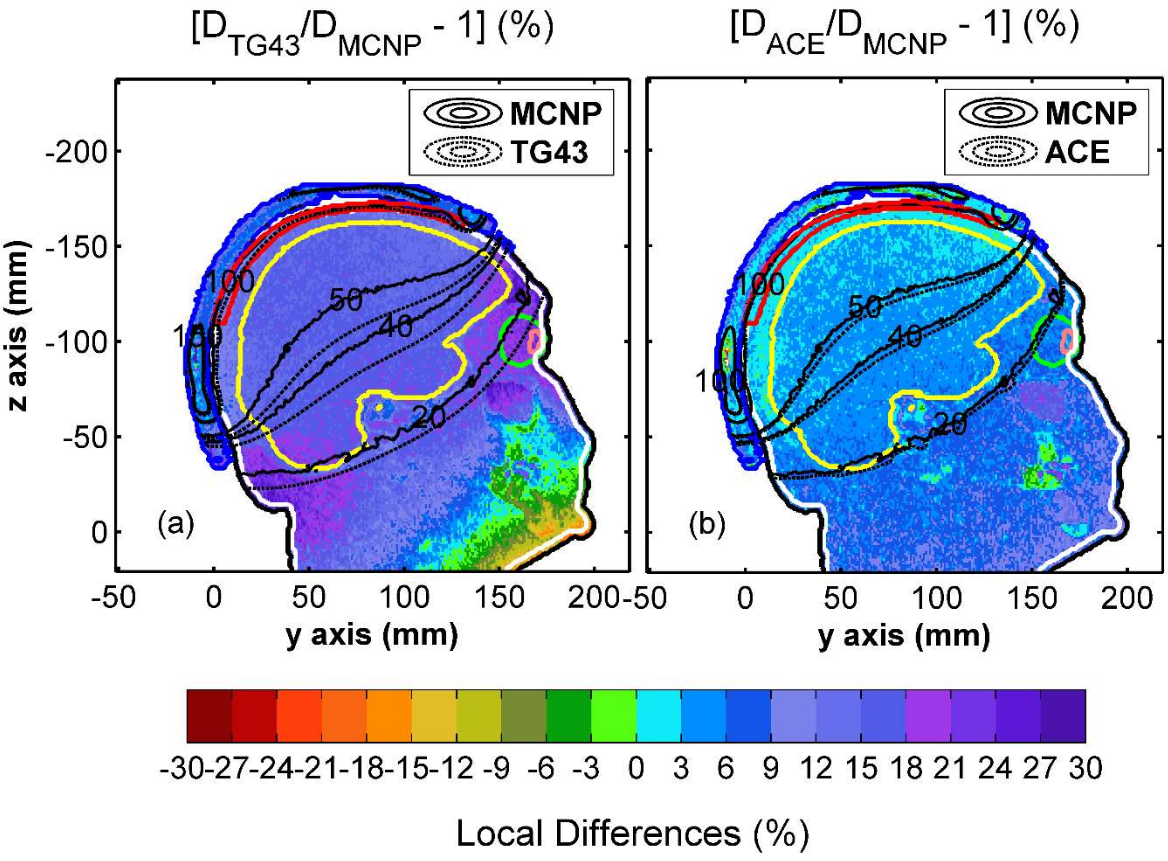

Fig. S2. Colormap representations of the local dose differences between (a) TG-43 and (b) HA ACE with corresponding MCNP results on a sagittal slice, with selected percentage isodose lines (20 %, 40 %, 50 %, 100 %, 150 %) superimposed (red contour: CTV, black contour: external, blue contour: mold, white contour: normal skin, yellow contour: brain, green contour: right eye, pink contour: right lens).

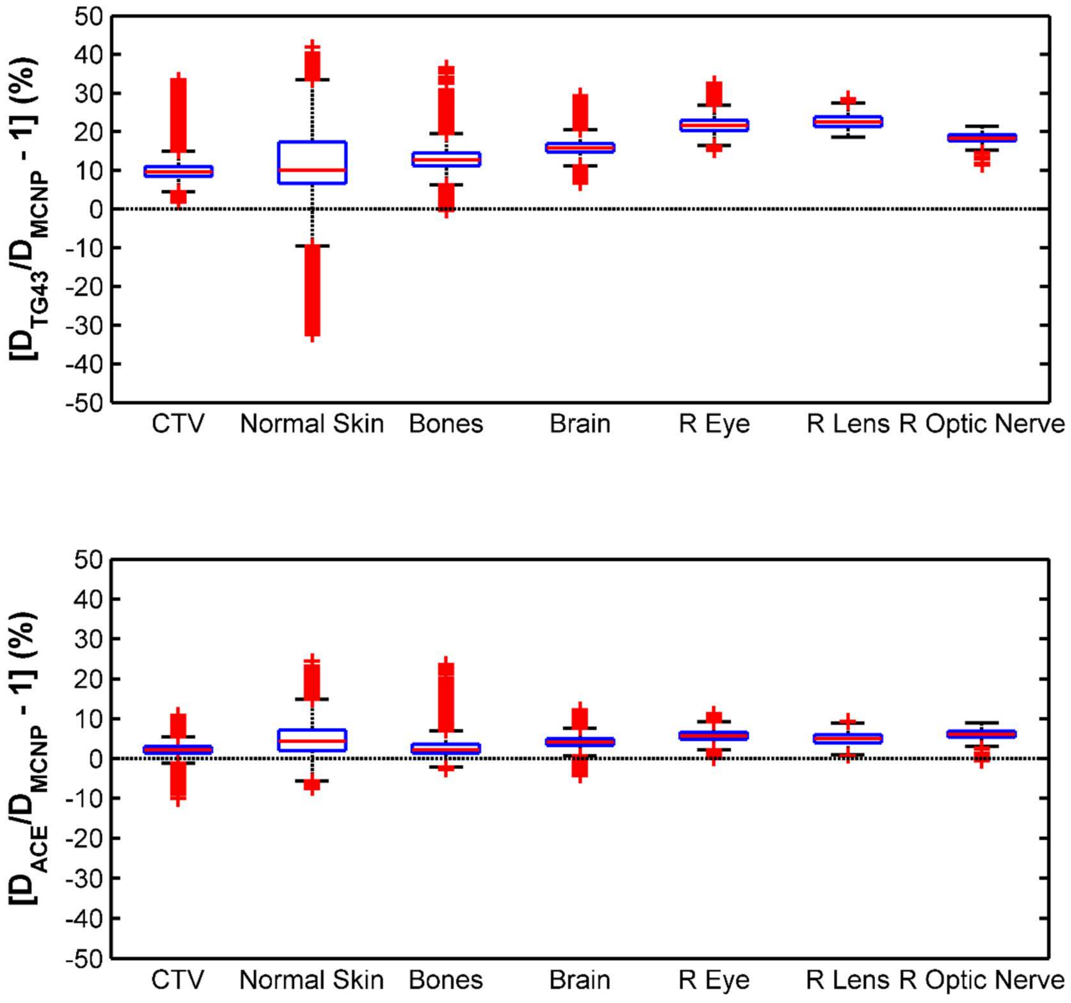

Fig. S3. Box and whiskers plots of the percentage dose differences between TG-43 (up) and HA ACE (down) with corresponding MCNP results, within the CTV, normal skin, bones, brain, right eye (R Eye), right lens (R Lens) and right optic nerve (R Optic Nerve). The red columns shown are formed by the overlapping of outliers.

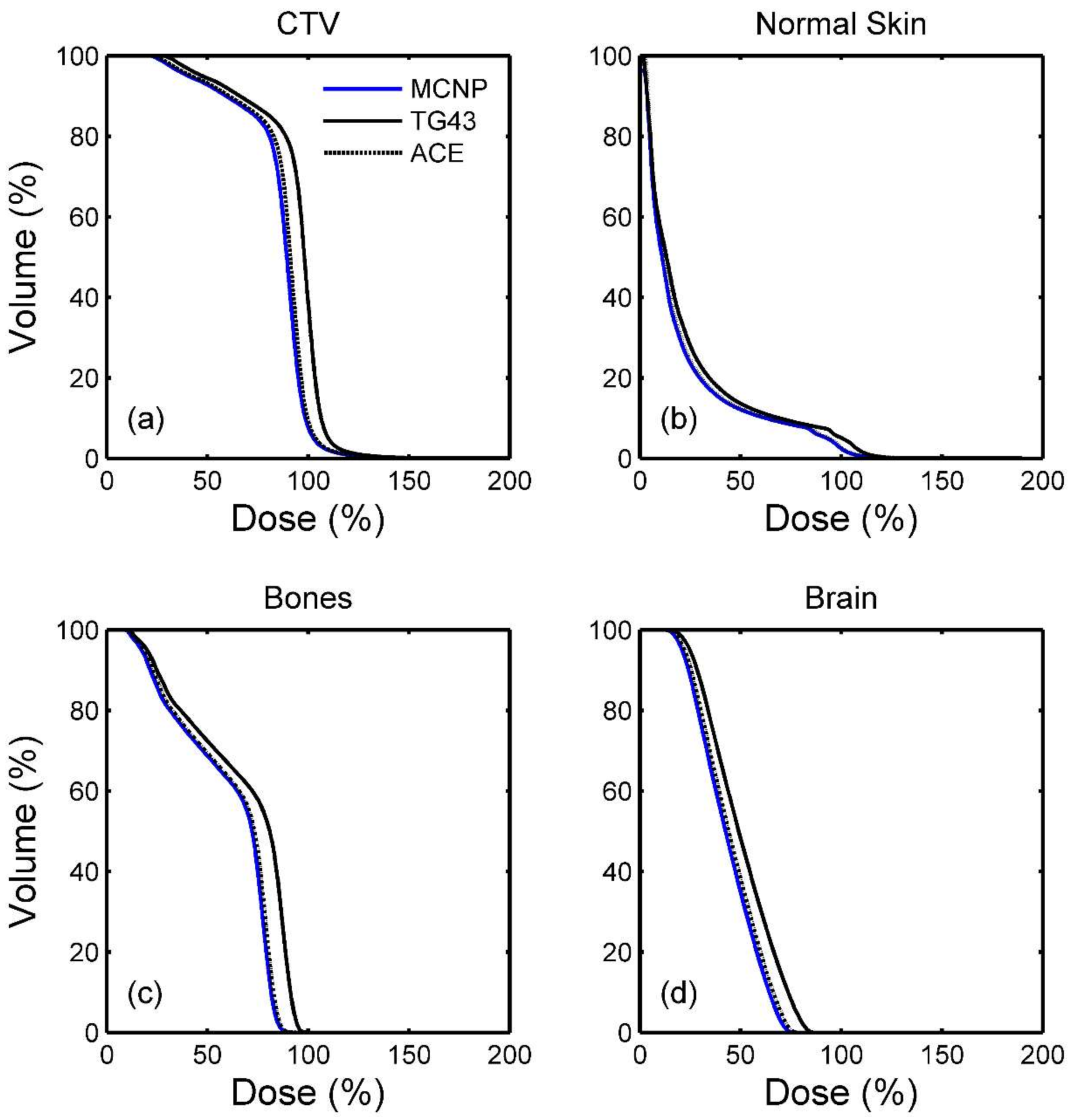

Fig. S4. DVHs for the (a) CTV, (b) normal skin, (c) bones, and (d) brain calculated using MCNP, TG-43 and HA ACE.

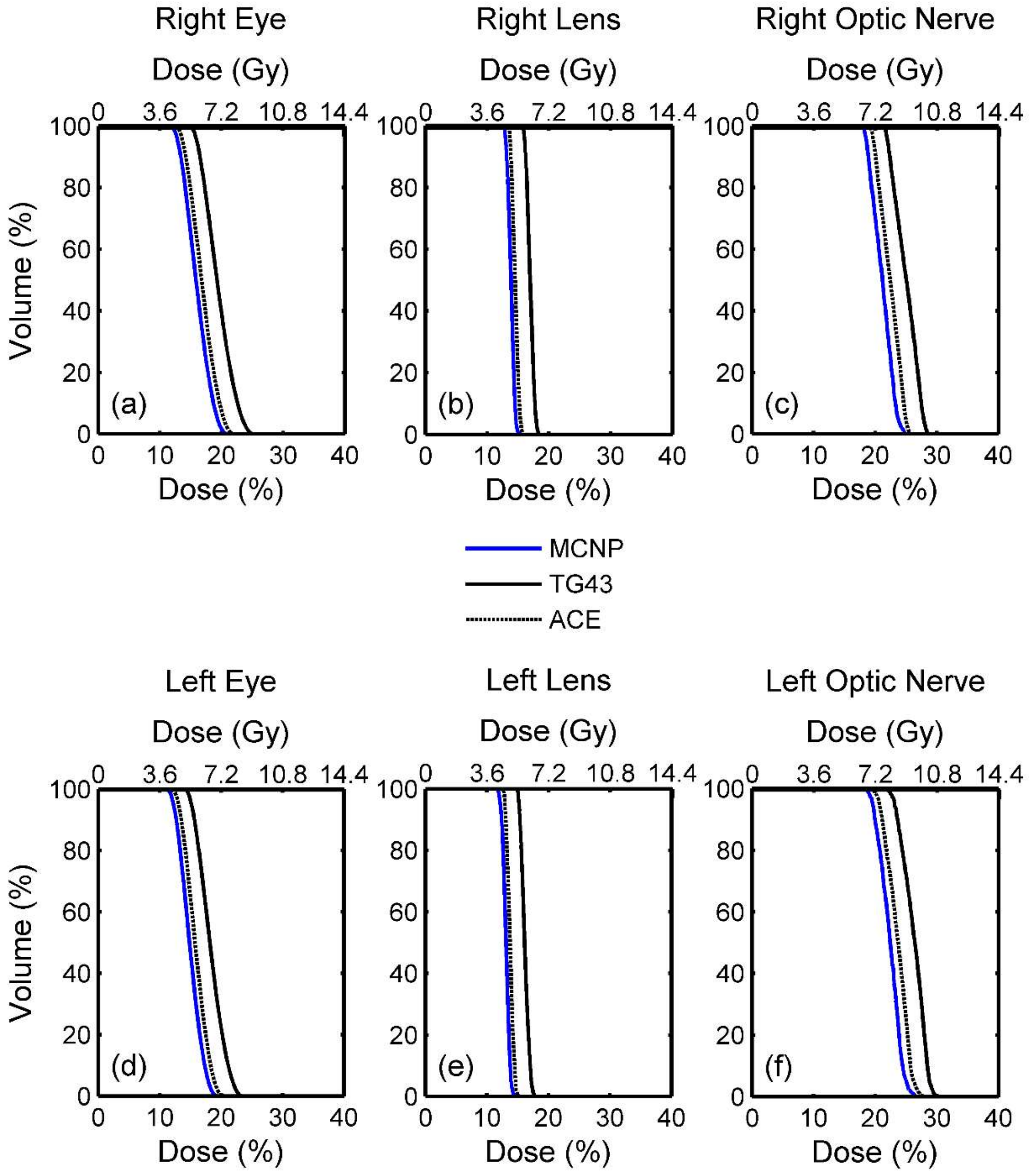

Fig. S5. DVHs for the (a) right and (d) left eyes, (b) right and (e) left lenses and (c) right and (f) left optic nerves calculated using MCNP, TG-43 and HA ACE.

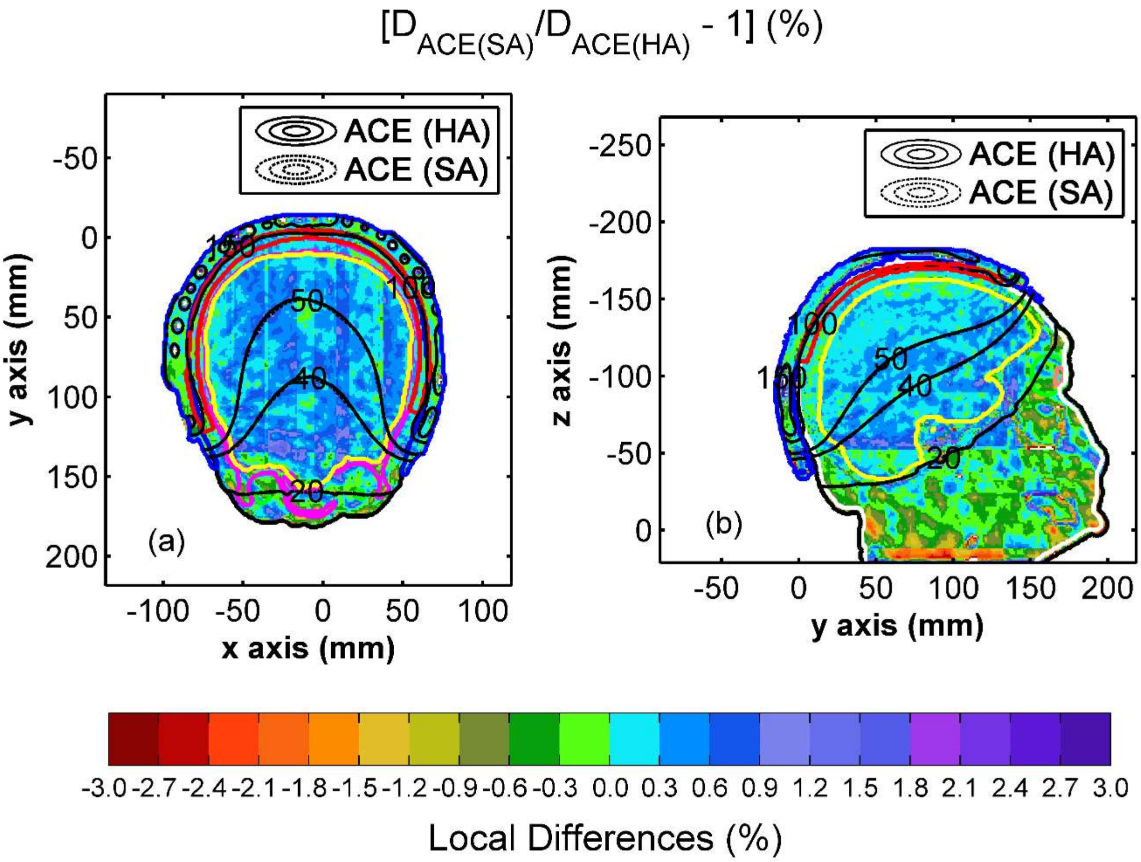

Fig. S6. Colormap representations of the local dose differences between SA and HA ACE on (a) the same axial slice as in Fig. 1, and (b) the same sagittal slice as in Fig. S2. Selected percentage isodose lines (20 %, 40 %, 50 %, 100 %, 150 %) are also superimposed (red contour: CTV, black contour: external, blue contour: mold, white contour: normal skin, yellow contour: brain, green contour: right eye, pink contour: right lens).

References

[1] Gorley JT, James MR, Booth TE, Brown FB, Bull JS, Cox LJ, et al. Initial MCNP6 Release Overview – MCNP Version 1.0. Los Alamos National Laboratory Tech. Rep. LA-UR-13-22934, Rev. 1. Los Alamos, NM, USA. June 2013.

[2] Pantelis E, Peppa V, Lahanas V, Pappas E, Papagiannis P. BrachyGuide: a brachytherapy-dedicated DICOM RT viewer and interface to Monte Carlo simulation software. J Appl Clin Medical Phys 2015;16:208-18. https://doi.org/10.1120/jacmp.v16i1.5136.

[3] Peppa V, Pappas EP, Karaiskos P, Major T, Polgár C, Papagiannis P. Dosimetric and radiobiological comparison of TG-43 and Monte Carlo calculations in ^{192}Ir breast brachytherapy applications. Phys Med 2016;32:1245-51. https://doi.org/10.1016/j.ejmp.2016.09.020.

[4] Schneider W, Bortfeld T, Schlegel W. Correlation between CT numbers and tissue parameters needed for Monte Carlo simulations of clinical dose distributions. Phys Med Biol 2000;45:459-78. https://doi.org/10.1088/0031-9155/45/2/314.

[5] Cullen DE, Hubbel JH, Kissel L. 1997. EPDL 97: The Evaluated Photon Data Library, `97 Version. Technical Report UCRL-50400, Vol 6, Rev 5, Lawrence Livermore National Laboratory, Livermore, CA. Available online at the IAEA website: https://www-nds.iaea.org/epdl97/. Last accessed March 2023.

[6] Shultis JK, Faw RE. An MCNP Primer. Kansas State University. Manhattan, KS, USA. 2011.
